# Targeting Mcl-1 by AMG-176 During Ibrutinib and Venetoclax Therapy in Chronic Lymphocytic Leukemia

**DOI:** 10.3389/fonc.2022.833714

**Published:** 2022-02-22

**Authors:** Xue Yi, Nitin Jain, LaKesla R. Iles, Mary L. Ayres, William G. Wierda, Varsha Gandhi

**Affiliations:** ^1^Department of Experimental Therapeutics, The University of Texas MD Anderson Cancer Center, Houston, TX, United States; ^2^Department of Leukemia, The University of Texas MD Anderson Cancer Center, Houston, TX, United States

**Keywords:** AMG-176, Bruton’s tyrosine kinase, chronic lymphocytic leukemia, ibrutinib, Mcl-1 protein, venetoclax

## Abstract

B-cell receptor (BCR) signaling pathway and Bcl-2 family prosurvival proteins, specifically Bcl-2 and Mcl-1, are functional in the pathobiology of chronic lymphocytic leukemia (CLL). A pivotal and apical molecule in the BCR pathway is Bruton’s tyrosine kinase (BTK). Together, BTK, Bcl-2, and Mcl-1 participate in the maintenance, migration, proliferation, and survival of CLL cells. Several ongoing and published clinical trials in CLL reported high rates of remission, namely, undetectable measurable residual disease (u-MRD) status with combined BTK inhibitor ibrutinib and Bcl-2 antagonist, venetoclax. While the majority of patients achieve complete remission with undetectable-measurable residual disease, at least one third of patients do not achieve this milestone. We hypothesized that cells persistent during ibrutinib and venetoclax therapy may be sensitive to combined venetoclax and Mcl-1 inhibitor, AMG-176. To test this hypothesis, we took peripheral blood samples at baseline, after Cycle 1 and Cycle 3 of ibrutinib monotherapy, after one week and 1 cycle of ibrutinib plus venetoclax therapy. These serial samples were tested for pharmacodynamic changes and treated *in vitro* with AMG-176 or in combination with venetoclax. Compared to C1D1 cells, residual cells during ibrutinib and venetoclax treatment were inherently resistant to endogenous cell death. Single agent exposure induced some apoptosis but combination of 100 nM venetoclax and 100 or 300 nM of AMG-176 resulted in 40–100% cell death in baseline samples. Cells obtained after four cycles of ibrutinib and one cycle of venetoclax, when treated with such concentration of venetoclax and AMG-176, showed 10–80% cell death. BCR signaling pathway, measured as autophosphorylation of BTK was inhibited throughout therapy in all post-therapy samples. Among four anti-apoptotic proteins, Mcl-1 and Bfl-1 decreased during therapy in most samples. Proapoptotic proteins decreased during therapy. Collectively, these data provide a rationale to test Mcl-1 antagonists alone or in combination in CLL during treatment with ibrutinib and venetoclax.

## Background

Survival, proliferation, migration, and maintenance of normal B cells and chronic lymphocytic leukemia (CLL) B cells are dependent on B-cell receptor (BCR) signaling pathway (1, 2). A pivotal molecule in the BCR signaling pathway is Bruton’s tyrosine kinase (BTK) which is at the apex of this nexus. Ibrutinib irreversibly inhibits BTK by covalently binding to the C481 residue of the kinase domain (3). The importance of BTK inhibition was clearly demonstrated by achievement of long-term progression-free and overall survival with ibrutinib monotherapy in patients with CLL versus standard chemotherapy or chemoimmunotherapy (4, 5). As a consequence, the drug was FDA approved as monotherapy for all CLL subgroups, namely, elderly patients (6) and patients with 17pdel (7). However, the complete remission rate was less than 5% in previously untreated patients with CLL (5).

In addition to the importance of BCR signaling pathway in the pathophysiology of CLL disease, Bcl-2 family prosurvival proteins (8, 9) were established as primary molecules responsible for apoptosis evasion of the malignant B cells. Among several genetic alterations in CLL, the most prevalent is deletion of chromosome 13q which occurs in 2/3 of patients (10). Identification of microRNA in this region and their role in overexpression of Bcl-2 and Mcl-1 further established role of these two antiapoptotic proteins in the biology of CLL (11, 12). Investigations of protein–protein interaction and fraction-based structural activity relationship for drug design were primary reasons for creation of navitoclax followed by venetoclax, a drug that exclusively neutralizes Bcl-2 antiapoptotic protein (13, 14). Its success as single-agent further established importance of Bcl-2 in the pathogenesis of CLL and especially in survival of malignant lymphocytes in patients. Venetoclax monotherapy results in 20% complete remissions and 5% undetectable measurable residual disease (u-MRD) status (15, 16). Hence, this remarkable pharmacological agent was also identified to be best used in mechanism-based combinations (17–19).

Both ibrutinib and venetoclax are approved, established, and effective single agents for treatment of CLL and other B-cell malignancies such as mantle cell lymphoma. However, in each case responses are mostly partial remissions; only a minority of patients achieve complete remissions with single agent treatments. Preclinically, we and others showed that levels of Mcl-1 (20) and Mcl-1 and BCL-xl (21) decreased after ibrutinib therapy resulting in priming of these cells for venetoclax-induced cytotoxicity. This decrease in survival proteins may be due to inhibition of BCR pathway and disruption of chemokine-controlled integrin-mediated homing of CLL cells (22). Murine models further provided evidence for benefits of combining BTK inhibitors with venetoclax (23, 24). Clinically, these drugs have non-overlapping toxicities, complementary yet distinct targets in CLL cells, and collective impact on CLL cells in all three niches such as blood, bone marrow, and lymph nodes (19). *Ex vivo* model that promotes CLL cell proliferation demonstrated that each drug works on different subpopulation resulting in synergy (25). Both drugs have been used in combination during clinical trials for B-cell malignancies such as mantle-cell lymphoma (MCL) (26), treatment-naïve CLL (18, 27, 28) or previously treated CLL (29, 30). Additional studies are further establishing clinical utility of the combination for CLL cells, namely, defined duration of therapy after achievement of u-MRD status (31, 32). Finally, results of the recent randomized GLOW trial demonstrated superior efficacy and safety of combined ibrutinib and venetoclax over chemoimmunotherapy (33). Pharmacodynamic endpoints during ibrutinib and venetoclax combination trials suggest 50–70% complete remission and u-MRD status in bone marrow or peripheral blood. Since all patients do not achieve CR or u-MRD status, these data suggest that there is/are additional molecules that provide survival advantage to CLL cells.

Mcl-1 was recognized among the top molecule in cancers for somatic copy number alterations (34). Mcl-1 protein levels have been shown to be important in the survival of CLL cells (20, 21, 35–41). Recently, direct inhibitors of Mcl-1 have been designed by Astra Zeneca [AZD-5991 (42)], Amgen [AMG-176 (43, 44)] and Novartis [S63845 and S64315 (45, 46)]. Clinical trials (NCT03218683; NCT02675452; NCT02992483) in liquid tumors are ongoing with these agents. Previously we established in preclinical studies that single-agent AMG-176 and S63845 were effective at inducing apoptosis in CLL cells (44, 47).

Based on this background, we hypothesized that AMG-176 will induce apoptosis in CLL cells previously treated with ibrutinib or combined ibrutinib and venetoclax therapy. To test this postulate, we took peripheral blood samples at baseline, after Cycle 1 and Cycle 3 of ibrutinib monotherapy, after one week of combination ibrutinib + venetoclax therapy, and after 1 complete cycle of combined ibrutinib and venetoclax. These serial samples were tested for pharmacodynamic changes and treated *in vitro* with AMG-176.

## Methods

### Clinical Trial

**Figure 1:**
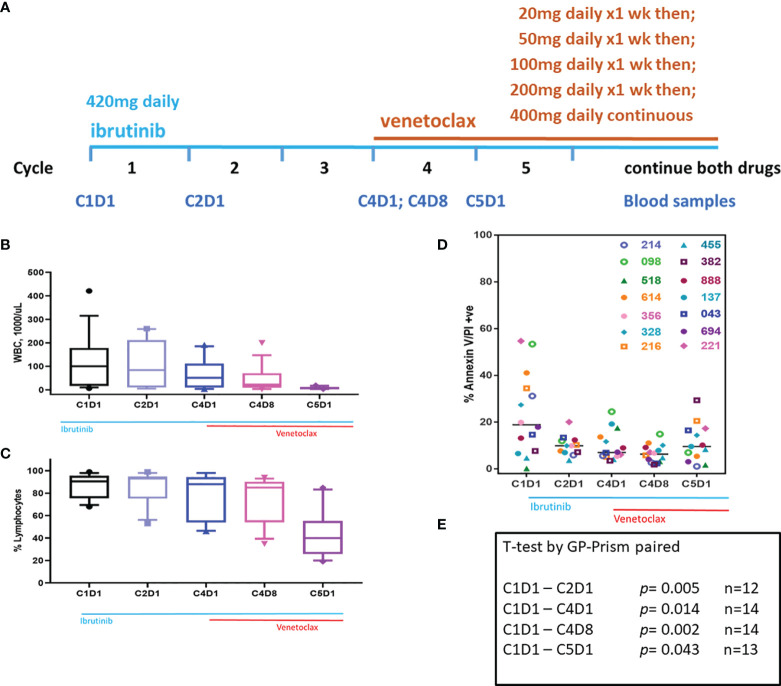
Clinical trial and changes in WBC, % lymphocytes, and sensitivity of blood cells for endogenous cell death in culture. **(A)** Clinical trial started with 3 cycles of ibrutinib monotherapy followed by ibrutinib/venetoclax couplet. Ibrutinib started at 420 mg/d while venetoclax had a ramp up dosing as indicated. Peripheral blood samples were taken at C1D1, C2D1, C4D1, C4D8, and C5D1. **(B)** White blood cells (WBC) count at baseline and changes in WBC during therapy. Box plots and whiskers represent 10–90 percentile. **(C)** Lymphocyte % at baseline and at different time points during therapy. Box plots and whiskers represent 10–90 percentile. **(D)** PBMCs isolated from peripheral blood samples were incubated for 24 h with DMSO and cell death was determined using flow cytometry after Annexin V/propidium iodide (PI) staining. Each symbol represents a patient. All patients were previously untreated except patients 518 and 888 who received one or two prior therapies, respectively. Horizontal black bars represent median values. **(E)** P-values were determined for each time point from the data in panel **(D)**. Number of patient samples at each time is provided. C, cycle and D, days.

The associated clinical trial was an investigator-initiated, open-label, phase II study of ibrutinib and venetoclax combination for patients with high-risk treatment-naive CLL or relapsed/refractory CLL (ClinicalTrials.gov number, NCT02756897). Patients initiated treatment with oral ibrutinib (420 mg once daily) as a single-agent for the first 3 cycles. The goals of ibrutinib monotherapy were to mobilize cells from lymph nodes, reduce tumor burden and consequently decrease the risk of tumor lysis syndrome (TLS), and to have CLL cells with lower level of Mcl-1 protein. After the 3 cycles of ibrutinib, venetoclax was initiated at the start of cycle 4 (**Figure 1A**). Oral venetoclax was dose-escalated in a weekly fashion (20 mg → 50 mg → 100 mg → 200 mg → 400 mg) to a target dose of 400 mg once daily, according to the prescribing information for venetoclax in CLL. Each cycle is four weeks. Combined venetoclax and ibrutinib were administered for a total of 24 cycles. Additional information regarding long-term treatment is provided in the original publication and in the recent update of the clinical trial results (18, 27).

### Patient Population

All patients in the study had an iwCLL treatment indication and were enrolled at The University of Texas MD Anderson Cancer Center (MDACC), Houston, TX, USA. Eligibility criteria were previously described in detail (18). Patients previously treated with ibrutinib or venetoclax were excluded. All patients were assessed for *IGHV* mutation status, chromosome abnormalities by fluorescence-*in-situ*-hybridization (FISH), and conventional cytogenetics. Targeted sequencing of 29 genes, including *TP53*, *SF3B1*, *NOTCH1*, *BIRC3*, *ATM*, was performed on tumor DNA from the pre-treatment BM aspirate.

### Patient Sample Collection and Processing

For pharmacodynamic investigations and *ex vivo* incubations with AMG-176 and/or venetoclax, serial peripheral blood samples were collected from patients during therapy. Peripheral blood was obtained from patients with CLL who provided written informed consent as part of a protocol approved by the Institutional Review Board of The University of Texas MD Anderson Cancer Center in accordance with the Declaration of Helsinki. Blood samples were obtained at different days (D) and cycles (C). Time points included pre-treatment (C1D1), after one cycle of ibrutinib monotherapy (C2D1), after 3 cycles of ibrutinib monotherapy (C4D1), after a week of combination ibrutinib + venetoclax therapy (C4D8), and after one cycle of the combination therapy (C5D1). Altogether, ~100 blood samples were obtained from 20 patients to be used for different experimental purposes. Baseline clinical and molecular characteristics of these patients are summarized in **Table 1** and described in the *Results* section.

**Table 1:** Patient characteristics.

ID	Sex	Age	Prior Rx#	Binet	Rai	PB- WBC	PB- HGB	PB- PLT	PB- Neut	PB- Lymph	PB- lgG	PB- lgA	PB- lgM	PB- LDH	PB_ B2M	IGHV	BM_Zap-70	FISH	Karyotype	TP53 mutation	Time to achieve U-MRD
888	F	62	2	B	I	12.4	11.4	137	9	85				566	3.5	UM	POS	11q	ND	NO	Never achieved
518	M	56	1	A	0	7.1	14.3	265	53	26	777	216	46	414	1.9	UM	POS	13Q	46,XY{20}	NO	9 mo COMBO
273	F	55	0	A	I	6.1	13.8	272	62	22				544	ND	UM	NEG	NEG	46,XX[20]	NO	12 mo COMBO
043	M	44	0	A	I	14.2	14.4	213	55	44	833	108	50	196	2.7	UM	POS	11Q	46,XY,del(11 )(q13q23)[4]/46,XY[16]	NO	9 mo COMBO
694	M	72	0	C	Ill	379	8.7	140	3	97	667	37	<11	474	5.2	UM	POS	NEG	46,XY[6]	NO	24 mo COMBO
328	M	74	0	A	I	11.1	13.6	128	35	63	677	154	67	471	4	UM	POS	17p	Complex	NO	3 mo COMBO
356	M	67	0	A	I	159	11.7	212	7	93	801	67	17	222	3.6	UM	POS	11Q	Complex	NO	18 mo COMBO
214	M	70	0	A	0	38.4	15.5	198	9	86				748	2.4	UM	NEG	T12	ND	NO	Never achieved
098	M	66	0	A	0	13.9	16.7	180	57	36	1347	9	39	538	1.9	MU	POS	T12	46,XY,+12[4]/47,idem,12;22)(q22;q13)[1]/46,XY[15]	NO	Never achieved
194	M	64	0	A	I	21.9	15.9	145	21	70	808	137	45	633	1.8	UM	POS	13Q	46,XY,t(13;17)(q14;q22X1]/46,XY[19]	NO	24 mo COMBO
749	M	84	0	C	Ill	102	9.3	119	5	90	979	269	22	216	3.7	UM	POS	11Q	ND	NO	Off study early due to MDS development
614	F	69	0	A	I	44.7	13.7	255	9	89	592	86	26	778	2.8	MU	NEG	13Q	46,XX[20]	NO	Never achieved
174	F	58	0	A	I	5.6	13	163	41	49	1151	296	137		2.6	UM	ND	17p	ND	YES	12 mo COMBO
213	M	59	0	A	I	221	14.2	142	5	94	1070	36	354	337	4	UM	POS	13Q	46,XY,del(6Xp21.3p23),ins(13;?)(q14;?)[4]/46,XY[16]	NO	3 mo COMBO
928	F	59	0	A	I	229	10.1	158	3	97	689	64	32	538	3.2	UM	POS	T12	47,XX,+12[6]/46,XX,[9]	NO	Off study early due to logistics
137	M	49	0	A	I	46.9	14.1	145	4	93	511	33	<11	652	2	UM	POS	NEG	ND	NO	18 mo COMBO
382	M	62	0	B	I	17.8	15.5	263	30	66				547	2.2	UM	POS	NEG	ND	NO	6 mo COMBO
455	M	61	0	C	IV	22.4	11.9	53	5	94	518	44	44	167	4.6	UM	POS	11Q	46,XY, deI(6)q13q23)[7]/46,XY[13]	YES	24 mo COMBO
216	M	57	0	A	0	65.7	14.3	206	8	89	821	58	21	559	2	UM	POS	NEG	47,XY,+6[1]/46,XY[19]	YES	3 mo COMBO
221	F	45	0	A	I	14.4	13.3	340	37	60	970	270	52	360	1.4	UM	POS	13Q	46,XX[20]	NO	12 mo COMBO

ID, unique identifier; WBC, White blood cells; HGB, hemoglobin; PLT, platelets; Neut, neutrophils; Lymph,% lymphocytes; LDH, lactate dehydrogenase; IGHV, immunoglobulin heavy chain; FISH, fluorescent in situ hybridization; UM, unmutated; MU, mutated; POS, positive; NEG, negative; M, male; F, female; 13Q,13q deletion; T12,trisomy 12;17p; 17p deletion;11q, 11q deletion; ND, not done; Rx, therapy; MDS, myelodysplastic syndrome; PB, peripheral blood; B2M, beta-2-microglobulin; u-MRD, undetectable measurable residual disease; mo, months; Combo, ibrutinib + venetoclax combined treatment.

In all cases, peripheral blood mononuclear cells (PBMCs) were isolated by Ficoll–Hypaque density centrifugation (GE Healthcare Bio-Sciences Corp., Piscataway, NJ) and suspended in Roswell Park Memorial Institute 1640 (RPMI 1640) media supplemented with 10% human serum (Sigma-Aldrich, St. Louis, MO). These samples were always used fresh for each experiment.

### Drugs

AMG-176 and venetoclax were purchased from Chemietek (Indianapolis, IN) and Xcessbio Company (San Diego, CA), respectively. For both drugs, stock solutions were made in dimethyl sulfoxide (DMSO; Sigma-Aldrich). All incubations were for 24 h at the indicated drug concentration alone or in combination. DMSO only was used as a vehicle control.

### *Ex Vivo* Drug Incubations

**Figure 2:**
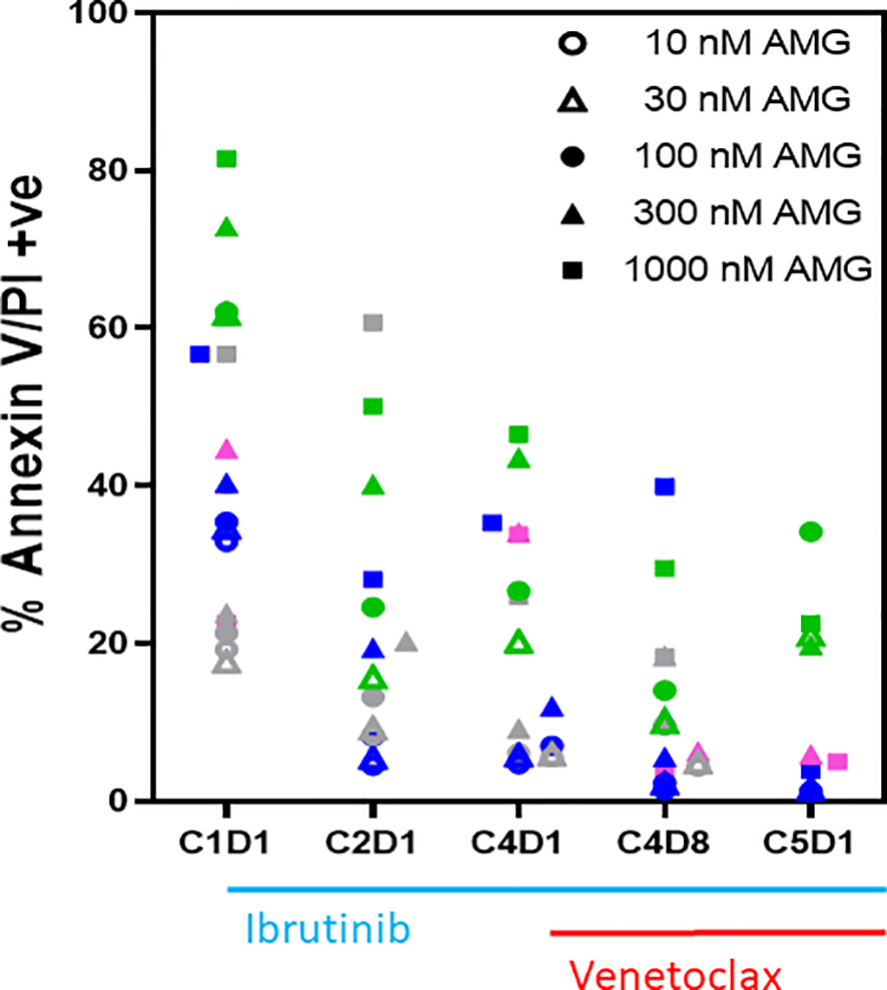
*Ex vivo* sensitivity of PBMCs to AMG-176 during ibrutinib monotherapy and ibrutinib plus venetoclax therapy. PBMCs from patients during therapy were obtained from peripheral blood. PBMCs from 4 patients were incubated *ex vivo* with 10–1,000 nM of AMG-176 for 24 h. Cell death was determined using flow cytometry after Annexin V/propidium iodide (PI) staining. Each color represents a patient and symbol denotes concentration of drug. Colors for symbols for patient 518, 356, 214, and 221 are pink, gray, blue, and green, respectively. AMG, AMG-176, C, cycle and D, days.

**Figure 3:**
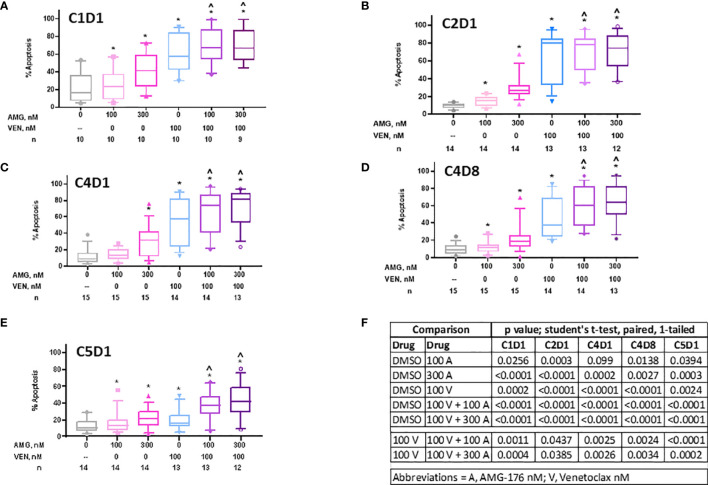
Sensitivity of PBMCs obtained during ibrutinib monotherapy and ibrutinib plus venetoclax therapy to AMG-176, venetoclax, or combination. PBMCs from patients during therapy were obtained and treated *ex vivo* with DMSO (0.1%), 100 nM AMG-176, 300 nM AMG-176, 100 nM venetoclax, or combination of AMG-176 at both concentrations with venetoclax. Cell death was determined using flow cytometry after Annexin V/propidium iodide (PI) staining. **(A)** PBMCs were obtained at baseline, i.e., C1D1. **(B)** PBMCs were obtained after one cycle of ibrutinib, i.e., C2D1. **(C)** PBMCs were obtained after three cycles of ibrutinib, i.e., C4D1. **(D)** PBMCs were obtained after addition of venetoclax for one week, i.e., C4D8. **(E)** PBMCs obtained after 4 cycles of ibrutinib and one cycle of venetoclax, i.e., C5D1. AMG, AMG-176, and VEN, venetoclax. Box plots and whiskers represent 10–90 percentile and horizontal line is median value. Number (n) of patients for each determination are listed under abscissa. **(F)** P-values were determined for different comparisons for data in panels **(A–E)**. * and ^ indicate p values <0.05 compared to DMSO or 100 V, respectively. C, cycle and D, days.

**Figure 4:**
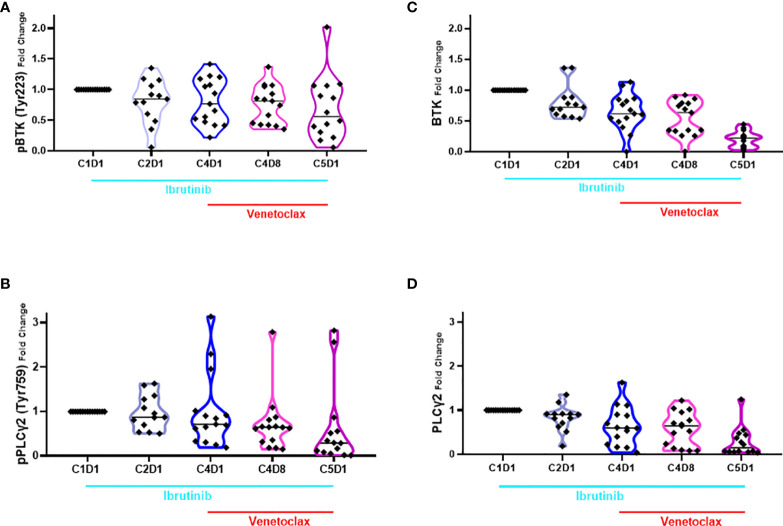
Violin plots for changes in the level of phospho- and total BTK and PLCγ2 proteins during ibrutinib monotherapy or ibrutinib plus venetoclax therapy. PBMCs from 13 to 15 patients were obtained at indicated times during therapy. Proteins were extracted and immunoblots were performed for pBTK **(A)**, pPLCγ2 **(B)**; total BTK **(C)** and total PLCγ2 **(D)**. Protein levels were quantitated and normalized with GAPDH and compared with the levels at baseline (C1D1). Each symbol represents a patient sample and horizontal lines are median values. Compared to C1D1 levels, the p-values for pBTK are 0.039, 0.032, 0.005, and 0.023 at C2D1, C4D1, C4D8, and C5D1. Compared to C1D1 levels, the p-values for pPLCγ2 are 0.418, 0.479, 0.048, and 0.072 at C2D1, C4D1, C4D8, and C5D1. Compared to C1D1 levels, the p-values for total BTK are 0.0113, 0.0002, <0.0001, and <0.0001 at C2D1, C4D1, C4D8, and C5D1. Compared to C1D1 levels, the p-values for total PLCγ2 are 0.0262, 0.0033, 0.0004, and <0.0001 at C2D1, C4D1, C4D8, and C5D1. C, cycle and D, days.

**Figure 5:**
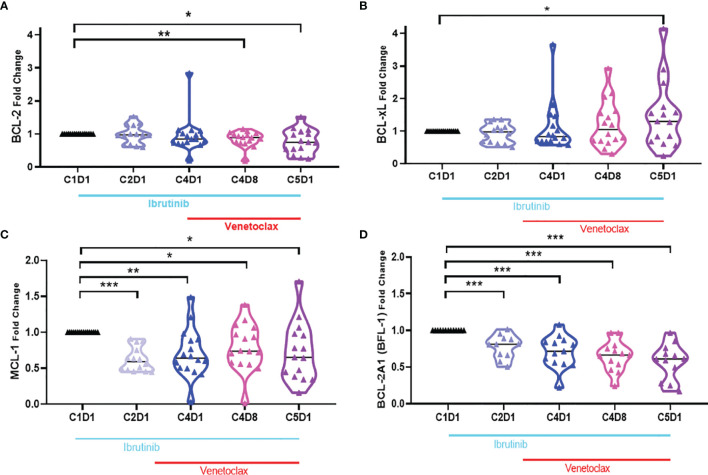
Violin plots for changes in the level of Bcl-2 antiapoptotic proteins during ibrutinib monotherapy or ibrutinib plus venetoclax therapy. PBMCs from 13 to 15 patients were obtained at indicated times during therapy. Proteins were extracted and immunoblots were performed for Bcl-2 **(A)**, Bcl-xL **(B)**, Mcl-1 **(C)**, and Bcl2A1 **(D)**. . Protein levels were quantitated and normalized with GAPDH and compared with the levels at baseline (C1D1). C, cycle and D, days. Statistical significance between any time points and baseline values is provided. *≤0.05; **≤0.001; ***≤0.0001.

**Figure 6:**
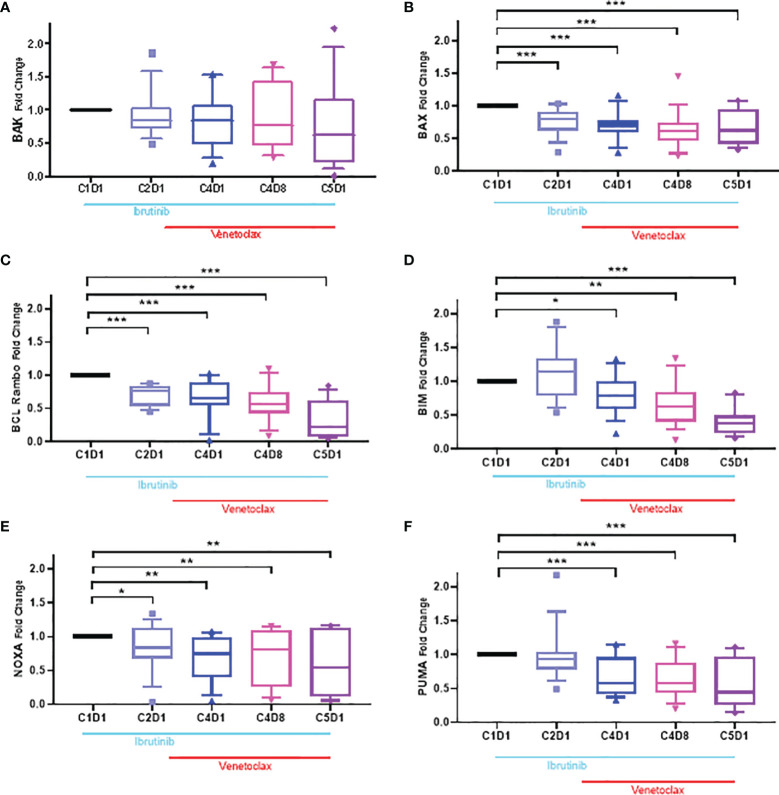
Box plots for changes in the level of Bcl-2 proapoptotic proteins during ibrutinib monotherapy or ibrutinib plus venetoclax therapy. PBMCs from 13 to 15 patients were obtained at indicated times during therapy. Proteins were extracted and immunoblots were performed for BAK **(A)**, BAX **(B)**; Bcl-Rambo **(C)** Bim **(D);** NOXA **(E)** and PUMA **(F)**. Protein levels were quantitated and normalized with GAPDH and compared with the levels at baseline (C1D1). Each box and whiskers represent 10 to 90 percentile and horizontal lines are median values. C, cycle and D, days. Statistical significance between any time points and baseline values is provided. *≤0.05; **≤0.001; ***≤0.0001.

CLL cells were isolated from the peripheral blood samples at 5 different time points and incubated *ex vivo* with vehicle (DMSO) only (**Figure 1**); AMG-176 at different concentrations (**Figure 2**); 100 nM AMG-176, 300 nM AMG-176, 100 nM venetoclax only; or combination of 100 nM venetoclax with 100 or 300 nM AMG-176 (**Figures 3**–**6**). All incubations were for 24 h.

### Cytotoxicity Assay

Cells were treated either with DMSO (vehicle control), or with single or combined drugs as indicated above for 24 h. For apoptosis assay, cells were stained with annexin V and propidium iodide (PI). Stained cells were analyzed by flow cytometry (BD Accuri C6, BD Biosciences). To calculate cell death, values from early apoptotic (Annexin^+^ PI^−^), late apoptotic (Annexin^+^ PI^+^) and necrotic (Annexin^−^ PI^+^) cells were included and labeled as % Annexin V/PI positive. Cells that were negative for both stains were considered live cells. Cell death of vehicle-treated samples was subtracted from drug-treated samples.

### Immunoblot Analyses

CLL cell pellets were suspended in modified RIPA buffer, with protease and phosphatase inhibitors, to extract total proteins. Whole cell lysates (20–30 µg) were used to perform the immunoblots and were run on Criterion protein gels (4–12% gradient gels; Bio-Rad). The gels were transferred onto Bio Rad immune-blot polyvinylidene difluoride (PVDF) membrane (Bio Rad #1620177), cut into slices, and incubated with specific antibodies, followed by visualization with the Odyssey Infrared Imaging System (LI-COR Biosciences, Lincoln, NE). Antibodies for specific proteins are listed in **Supplementary Table 1**.

### Quantitation of Immunoblots

The signal intensity of each band of target protein and also internal control (either Vinculin or GAPDH) was measured using Licor Image Studio software following manufacturer’s guideline (www.Licor.com). Values were normalized using loading control and then were compared to that in vehicle control. Final data were plotted into GraphPad Prism 7 for representation.

### Statistical Analysis

All graphs were created using Prism software (GraphPad). To determine statistical significance, Prism software was used and samples in the control (DMSO treated) were compared with drug treated cells using one-tailed paired Student’s t-test. P-values lower than 0.05 were considered significant. Values are provided either in the figure legend, included in the figure as a table, or included in the figure as an asterisk.

## Results

### Patient Characteristics

Sample collection, drug doses, and protocol schema are provided (**Figure 1A**). Characteristics of patients (n = 20) is described (**Table 1**). Majority of patients were treatment naïve. All except 2 patients had unmutated IGHV. ZAP-70 status, B2M level, hematological data, and CLL disease staging are listed in **Table 1**. All patients had at least one of the five feature: del(17p), mutated *TP53*, del(11q), unmutated *IGHV*, or age ≥65 years. Unique patient identifiers are provided in the first column and these numbers are listed in the figures.

### Inherent Capability of Cell Death

During therapy, WBC count in peripheral blood decreased after ibrutinib monotherapy and, in particular, after the addition of venetoclax (**Figure 1B**). Lymphocyte count in the peripheral blood was similar for the first four time points but decreased at C5D1 which likely reflects venetoclax-induced apoptosis (**Figure 1C**). When grown in culture in regular medium (containing 10% human serum), CLL lymphocytes undergo cell death in a time-dependent manner. This endogenous cell death is heterogeneous among patients. To determine if inherent cell death would be different after treatment with ibrutinib or ibrutinib plus venetoclax, pretreatment and post-therapy samples were cultured in DMSO (vehicle control) for 24 h. Baseline (pretreatment) sample showed variation in endogenous cell death ranging from 0 to 55%. Compared to this baseline (C1D1) sample, paired samples at different time points showed much less cell death (**Figure 1D**). The values were significantly different in paired samples at every time point starting with C2D1 and ending with C5D1 (p = 0.04 to 0.002; **Figure 1E**). These data suggest that persister cells in the peripheral blood after treatment with ibrutinib alone or in combination with venetoclax were relatively resistant to inherent cell death.

### Sensitivity of CLL Lymphocytes to AMG-176 During Ibrutinib Monotherapy and Ibrutinib Plus Venetoclax Therapy

To select a dose of AMG-176 and to test if CLL lymphocytes show more or less sensitivity to AMG-176 after treatment with ibrutinib or combination ibrutinib/venetoclax, four patient samples (#518, 356, 214, 221) were used for this investigation; each patient is represented by a color which is defined in the figure legend (**Figure 2**). Similar to endogenous cell death data, there was heterogeneity for cell death among baseline (C1D1) samples, yet there was evidence of some dose-dependency. However, in most cases, the extent of cell death with exogenous AMG-176 decreased after treatment with ibrutinib or combination (C4D8 and C5D1). These data suggest that not only do lymphocytes show relatively less sensitivity to endogenous cell death, they were less sensitive to AMG-176-induced cell death also after ibrutinib or ibrutinib plus venetoclax therapy. Based on dose-dependency (**Figure 2**) and achievable plasma concentrations, we selected 100 and 300 nM AMG-176 for future combination experiments presented in **Figure 3**.

### Sensitivity of CLL Lymphocytes to AMG-176, Venetoclax, or Their Combination During Ibrutinib Monotherapy and Ibrutinib Plus Venetoclax Therapy

We evaluated if peripheral blood cells at baseline (C1D1), during ibrutinib alone (C2D1, C4D1), or ibrutinib and venetoclax (C4D8 and C5D1) were sensitive to additional *ex vivo* incubations with 100 nM and 300 nM AMG-176, 100 nM venetoclax, or venetoclax in combination with 100 nM or 300 nM AMG-176 (**Supplementary Figure 1** and **Table 2**). Cell death data from 10 to 15 patients (numbers under abscissa) are presented in separate graphs for each time point (**Figures 3A–E**) and data for each drug alone and in combinations were compared with DMSO for each time point with statistical analyses or venetoclax alone was compared with combination (**Figure 3F**).

**Table 2:** Percent cell death in PBMCs obtained during ibrutinib and venetoclax trial after *ex vivo* incubations with AMG-176 alone, venetoclax alone, or both drugs in combination.

Time Point →	% Cell death, Mean ± SEM				
Drug, conc ↓	C1D1	C2D1	C4D1	C4D8	C5D1
A 100 nM	25.7 ± 5.7	14.9 ± 1.6	14.1 ± 1.9	12.3 ± 1.6	16.5 ± 3.6
A 300 nM	41.9 ± 7.0	30.0 ± 3.6	30.9 ± 4.8	23.1 ± 4.5	21.8 ± 3.3
V 100 nM	60.8 ± 6.9	64.5 ± 7.8	54.2 ± 7.2	45.3 ± 6.3	19.7 ± 3.5
V 100 nM + A 100 nM	69.8 ± 6.2	70.6 ± 5.8	67.1 ± 6.9	59.9 ± 6.2	36.8 ± 4.9
V 100 nM + A 300 nM	69.7 ± 6.3	70.2 ± 6.1	70.6 ± 6.3	64.4 ± 6.2	42.5 ± 6.2

C, cycle; D, day; A, AMG-176; V, venetoclax.

Prior to any therapy (i.e., C1D1 time point) cells were sensitive to AMG-176 alone (Mean 26% and 42% cell death, p = 0.026 and <0.0001 at 100 and 300 nM, respectively), venetoclax alone (Mean 61% cell death, p = 0.0002) and combination resulted in similar cell death as venetoclax alone (mean 70% cell death with p = <0.001 at both AMG-176 concentrations) (**Figures 3A**, **3F** and **Table 2**).

Cells from patients receiving ibrutinib monotherapy (C2D1 and C4D1) showed a mean 64.5% ± 7.8% and 54.1% ± 7.2% venetoclax-induced cell death (p = <0.0001 at both time points). Moderate yet significant increase in cell death was demonstrated when 100 nM (70.6% ± 5.8% and 67.1% ± 6.9% cell death; p = 0.04 in C2D1 and 0.002 in C4D1 sample) or 300 nM AMG-176 (70.2% ± 6.1% and 70.6% ± 6.3%; p = 0.04 in C2D1 and 0.002 in C4D1 sample) was added exogenously (**Figures 3B, C, F**, **Table 2**).

Cells taken from patients after 3 cycles of ibrutinib and one week of ibrutinib plus venetoclax (at 20 mg daily dose; C4D8; **Figure 1A**) showed less cell death compared to C2D1 and C4D1 samples (ibrutinib monotherapy) with *ex vivo* incubations with each drug alone or in combination (**Table 2**). However, compared to venetoclax alone (45.3% ± 6.3%), these cells had higher level of apoptosis when both drugs (AMG-176 and venetoclax) were added exogenously (59.9% ± 6.3% p = 0.002 at 100 nM and 64.4% ± 6.2%, p = 0.003 at 300 nM AMG-176) (**Figure 3D** and **Table 2**). Similarly, at C5D1 time point (with 4 cycles of ibrutinib and with 4 weeks of ramp-up dosing of venetoclax; **Figure 1A**), compared to venetoclax alone (19.7% ± 3.5%), the combination of venetoclax and 100 nM AMG-176 (36.8% ± 4.9%, p ≤0.0002) or 300 nM AMG-176 (42.5% ± 6.2%, p = <0.0002) resulted in increased cell death (**Figure 3E** and **Table 2**).

### Inhibition of the BCR Pathway Signaling During Ibrutinib Monotherapy and Ibrutinib Plus Venetoclax Therapy

The action of ibrutinib is on phosphorylation of BTK and, by extension, inhibition of the BCR signaling pathway while venetoclax targets Bcl-2, one of the most abundant antiapoptotic proteins in CLL lymphocytes. Hence, in 16 patient samples where enough cells were available, we performed immunoblot assay for BCR transduction proteins and Bcl-2 pro- and anti-apoptotic proteins (**Supplementary Figure 2**). The protein levels were quantitated, normalized with loading control, and expressed as percent of baseline (**Supplemental Figure 3**, **4A–C**). Among the BCR signaling pathway proteins, BTK and PLCγ2 were evaluated; statistical values are provided in the figure legend (**Figure 4**). Decrease in phospho-BTK levels was observed starting with C2D1 and further declined with additional treatment (**Figure 4A**). For phospho-PLCγ2, there was a trend in a mean decrease in the value, but this was only significantly different for C4D8 (**Figure 4B**). Median value of phospho-PLCγ2 at Tyr1217 remained similar at different time points and was not significantly different (**Supplementary Figure 5**); of note, this site is not phosphorylated by BTK. Previously we showed that total BTK transcript and total protein levels decrease in CLL cells during ibrutinib therapy (48). This was also observed in the current therapy and was significantly different at all time points compared to pretreatment value (**Figure 4C**). Similarly, significant decline in total PLCγ2 was observed at all time points compared to baseline value (**Figure 4D**).

### Changes in Bcl-2 Antiapoptotic Family Proteins During Ibrutinib Monotherapy and Ibrutinib Plus Venetoclax Therapy

Four of the 6 members of the Bcl-2 family of anti-apoptotic proteins were detected in CLL lymphocytes (**Supplementary Figures 3****,** **4** and **Figures 5A–D**). Bcl-2 protein showed heterogeneous response among patients treated with ibrutinib which was not significantly different than the baseline value. However, after addition of venetoclax, the levels of Bcl-2 proteins (**Figure 5A**) were significantly different at C4D8 (p = 0.0095) and C5D1 (0.0160). Bcl-XL protein levels (**Figure 5B**) were not significantly changed, although there was a trend for an increase in some patient samples (C5D1; p = 0.0463). In contrast to Bcl-2 and Bcl-XL, compared to C1D1 values, aggregate values for Mcl-1 and Bfl-1 proteins (**Figures 5C, D**) continuously and significantly decreased at all four time points (p = 0.0103 − <0.0001). However, regarding Mcl-1, there is heterogeneity among 16 patient samples (**Supplementary Figure 6**). For the 4 patients, who did not achieve u-MRD, Mcl-1 levels remained unchanged in patient 214; decreased precipitously in patient 888; decreased first and then increased in patients 098 and 614. CLL cells after ibrutinib treatment had heterogeneous response to uncleaved PARP protein, which was not statistically significantly different, however, after addition of venetoclax (C4D8 and C5D1), there was a significant decline in PARP protein indicating induction of apoptosis after addition of venetoclax (**Supplementary Figure 7**).

### Changes in Bcl-2 Proapoptotic Family Proteins During Ibrutinib Monotherapy and Ibrutinib Plus Venetoclax Therapy

There are several members of the Bcl-2 family proapoptotic proteins. Six of these proteins were tested in CLL lymphocytes (**Supplementary Figure 4**). Bax and Bak are multidomain proapoptotic Bcl-2 family proteins needed for intrinsic cell death (49). Levels of Bak showed some perturbations but generally the changes were not significant (p-value, 0.05–0.23). In contrast, level of Bax protein showed a 50% decline which was consistent in all 4 time points after start of ibrutinib and ibrutinib plus venetoclax therapy (**Figures 6A, B**). Four different BH3-only domain Bcl-2 family proteins were also tested. Bcl-2 Rambo levels decreased in all patients (p ≤0.0004), however, the extent was heterogeneous among patients (**Figure 6C**). Bim protein (**Figure 6D**) had heterogeneous and non-significant (p = 0.123) response after one cycle of ibrutinib but then declined at other time points (p = 0.011 − <0.0001). Compared to baseline value in C1D1 samples, both Noxa and Puma proteins (**Figures 6E, F**) were significantly reduced after start of ibrutinib and decreased further after addition of venetoclax (p = 0.03 − <0.0001).

## Discussion

Our current investigations suggest that peripheral blood cells that persist after a few cycles of ibrutinib and one cycle of venetoclax therapy are inherently less primed for apoptosis. While they do not show high sensitivity to either AMG-176 or venetoclax alone, they are sensitized *ex vivo* to treatment with combined Mcl-1 and Bcl-2 antagonists, AMG-176 and venetoclax.

Pharmacodynamic investigations during ibrutinib monotherapy showed decline in Mcl-1 protein levels with no or limited changes in the level of Bcl-2 protein (20). We observed similar changes in the level of Mcl-1 protein with another covalent BTK inhibitor, acalabrutinib (50, 51) and was also reported by another group (24) indicating the role of the BCR signaling pathway in this pharmacodynamic effect. Consistent with this postulate, PI3 kinase inhibitors such as idelalisib (52) and duvelisib (53) incubations or therapy resulted in modulation of Mcl-1 protein levels. Our present investigations in ibrutinib followed by ibrutinib and venetoclax couplet clinical trial patient samples further establish significant reduction in Mcl-1 protein (**Figure 5C**). Previously we have demonstrated in CLL cells that AMG-176-mediated CLL cell death was inversely proportional to level of Mcl-1 protein (44). The relevant and unanswered question with this regard is whether the Mcl-1 protein can be targeted by direct antagonist such as AMG-176 to sensitize CLL cells that remain during treatment with ibrutinib and venetoclax.

AMG-176 is a potent antagonist of Mcl-1; in cell free system the Ki value is <1 nM. In whole cells, the drug was tested in different leukemias, lymphoma, and multiple myeloma cell lines (43). Pertaining to current work, AMG-176 was tested in primary CLL cells from 74 patient samples showing cell death when 300 nM concentration was used (44). We also observed an inverse correlation of AMG-176 induced cell death and endogenous levels of Mcl-1. Considering this relationship, ibrutinib-mediated decrease in Mcl-1 provides a rationale to combine AMG-176 during or after combined treatment with ibrutinib and venetoclax.

The primary target of ibrutinib is BTK which is critical in the BCR signaling pathway. Its centrality in the BCR signaling pathway and maintenance of B-cells became clear with the therapeutic success of BTKi (54). Pharmacodynamic analysis of BCR signaling using phospho-BTK protein as a biomarker clearly demonstrated that in circulating CLL cells of the 16 patients evaluated, this pathway was hampered during the four cycles of ibrutinib monotherapy. In addition to a decrease in phospho-BTK, total BTK protein (**Figures 4A, B**) was also diminished which is consistent with our prior observations (48, 55).

The current project did not focus on mechanism of Mcl-1 decline after ibrutinib therapy and collateral induction of apoptosis. Somatic copy number alteration studies in many cancers identified Mcl-1 as the topmost genes with increased copy number (34). It is also highly expressed in many human cancers (56). Specific to CLL, this prosurvival protein was shown to be essential during early lymphoid development. Furthermore, maintenance of mature T and B-lymphocytes is dependent upon this protein (57). These observations underscore centrality of this protein in lymphocytes and suggest demise of these cells when Mcl-1 dissipates. However, it is not clear how this protein declines with ibrutinib therapy. Activation of the BCR signaling pathway leads to activation of several transcription factors, which may be responsible for transcription of *MCL*-1 gene. Further, Mcl-1 is a short-lived protein and diminishes with a half-life of <1 h (58). Additionally, onset of apoptosis of leukemia cells can further degrade Mcl-1 as this protein has two caspase cleavage sites. Once cleaved, the C-terminal domain of Mcl-1 acts as a pro-apoptotic molecule after caspase-3 cleavage which pushes intrinsic apoptosis (59). Caspase-mediated cleavage and dissipation of Mcl-1 may be a highly likely scenario in CLL cells from patients treated with ibrutinib and venetoclax therapy, however, such postulates need to be tested.

Bcl-2, Mcl-1, Bfl-1, and Bcl-XL are four primary antiapoptotic molecules of the Bcl-2 family survival proteins that are expressed in CLL lymphocytes. Their abundance results in evasion of apoptosis in the malignant lymphocytes. While Bcl-2 and Mcl-1 are being antagonized with our current approach in the present paper, Bfl-1 and Bcl-XL remain untargeted. Bfl-1 protein, however, was consistently and continuously decreased during therapy while Bcl-XL levels were significantly induced, especially at the C5D1 time point (**Figure 5B**). Further, there appears to be heterogeneity and 50% of the samples showed a high increase in Bcl-XL. It is possible that circulating CLL cells with high level of Bcl-XL are being enriched after *in vivo* treatment with ibrutinib and venetoclax. On the other hand, mimicking *in vivo* environment during *in vitro* investigations in CLL and mantle cell lymphoma cells also showed Mcl-1, Bcl-XL, and survivin as primary determinants of ibrutinib and venetoclax resistance (41). Collectively, these data also provide rationale to test Bcl-XL inhibitors after combined BTKi and venetoclax.

Several investigator-initiated trials and multicenter studies have now established clinical success of ibrutinib and venetoclax combination in CLL and also in mantle cell lymphoma (26). One of the first trials of this couplet in CLL was conducted at our center and reported high rates of complete remission, undetectable measurable residual disease (u-MRD), overall survival and progression-free survival in previously untreated patients with poor prognosis attributes (18). Impressively, high remission, survival, and u-MRD status were maintained even after three years of therapy which included one year without treatment in patients who had u-MRD or with ibrutinib maintenance therapy for patients who had detectable measurable disease (27). CAPTIVATE study further attested superiority of this combination resulting in the 30-month progression free survival rate of ~95% across all treated patients. Similar data are emerging from many different institutes in large studies (28, 29) and also for patients with relapsed/refractory CLL (29, 30, 60). Recent results from the randomized trial of ibrutinib and venetoclax versus chlorambucil and obinutuzumab (CLL GLOW trial) validated the ibrutinib and venetoclax couplet therapy for CLL as a time-limited oral targeted therapy for patients with CLL (33). Collectively, these data demonstrate high u-MRD in both untreated and previously treated CLL but also emphasize that there are patients who do not respond to this combination, underscoring a need for next step. Our current data clearly provide evidence in primary target cells during the ibrutinib and venetoclax couplet therapy. Our pharmacokinetic profiling and pharmacodynamic investigations demonstrate Mcl-1 inhibitors such as AMG-176 as a suitable partner with ibrutinib and venetoclax.

All three direct inhibitors of Mcl-1, e.g. AMG-176, have been tested in the clinic for hematological malignancies. However, untoward toxicities of these agents have hampered the enthusiasm. Modification of these drugs and structure activity relationship may result in a better clinical candidate. Additionally, new schedules are being tested in the clinic trials which may help ameliorate toxicity. An indirect approach to target Mcl-1 is to transiently inhibit transcription or protein translation of this anti-apoptotic protein using transcription and translation inhibitors. Mcl-1 has short transcript turn-over and short protein half-life suggesting transcription and translation inhibitors as an option to reduce levels of Mcl-1. CDK9 and CDK7 are key kinases that phosphorylate serine residues on the heptamer repeats on the C-terminal of RNA polymerase II which is critical for mRNA syntheses (61). Flavopiridol and SNS-032 are potent inhibitors of CDK7 and 9; treatment with these agents decreased levels of short-lived transcripts such as Mcl-1 and Myc. Such strategies have been successful both during *in vitro* incubations and during therapy for CLL (62, 63), and Mcl-1 was predictor of cell death (64). Because Mcl-1 protein half-life is short, inhibition of translation could also play a role in dissipation of Mcl-1 protein and induction of apoptosis. Clinical candidates such as omacetaxine and silvestrol, both natural products, block protein synthesis and were utilized as protein translation inhibitor (65, 66). These transcription and translation inhibitors need to be tested on persister cells after combined ibrutinib and venetoclax therapy.

Taken together, our data provide evidence for additional combination strategies with ibrutinib and venetoclax for patients with CLL. Because direct and indirect antagonists of Mcl-1 are available for clinical use, such approach could be translated to clinic for patients with B-cell malignancies in general and CLL in particular.

## Data Availability Statement

The original contributions presented in the study are included in the article/**Supplementary Material**. Further inquiries can be directed to the corresponding author.

## Ethics Statement

For blood sample collections, patient provided written informed consent for protocols approved by the Institutional Review Board of The University of Texas MD Anderson Cancer Center, in accordance with the Declaration of Helsinki. The patients/participants provided their written informed consent to participate in this study.

## Author Contributions

XY designed and performed the experiments and analyzed the results. NJ was Principal Investigator for clinical trial and directed patient sample collection, provided clinical and patient-related input, and reviewed the manuscript. LI performed all immunoblots and assisted in graphing data. MA coordinated blood sample collection from patients and processed samples. WW was Co-principal Investigator of the clinical trial, identified patients to obtain peripheral blood samples, provided clinical and patient-related input, and reviewed the manuscript. VG was Co-principal Investigator of the clinical trial, conceptualized and supervised current research project, obtained funding, analyzed the data, and wrote and reviewed the manuscript. All authors listed have made a substantial, direct, and intellectual contribution to the work and approved it for publication.

## Funding

This work was supported in part by a CLL Global Research Foundation Alliance grant and MD Anderson’s CLL Moon Shot™ program. XY received scholarship from China to work as a visiting scientist.

## Conflict of Interest

NJ has Consultant/Honoraria: Pharmacyclics, ADC Therapeutics, Adaptive Biotechnologies, AbbVie/Genentech, Janssen, AstraZeneca/MedImmune, Servier, Precision, Biosciences, BeiGene, TG Therapeutics, Cellectis, Bristol Myers Squibb/Celgene, Research Funding: Pfizer, Pharmacyclics, AbbVie, Genentech/Roche, Incyte, Infinity Pharmaceuticals, Bristol Myers Squibb, Seattle Genetics, Celgene, ADC Therapeutics, Servier, AstraZeneca/MedImmune, Cellectis, Adaptive Biotechnologies, Precision Biosciences, Aprea Therapeutics, fate therapeutics, Kite. WW has received research funding from GSK/Novartis, Abbvie, Genentech, Pharmacyclics LLC, AstraZeneca/Acerta Pharma, Gilead Sciences, Juno Therapeutics, KITE Pharma, Sunesis, Miragen, Oncternal Therapeutics, Inc., Cyclacel, Loxo Oncology, Inc., Janssen, Xencor. VG has received research funding from Acerta Pharma, AbbVie, Clear Creek Bio, Gilead Sciences, Infinity, Loxo Oncology, Pharmacyclics, and Sunesis.

The remaining authors declare no competing financial interests.

## Publisher’s Note

All claims expressed in this article are solely those of the authors and do not necessarily represent those of their affiliated organizations, or those of the publisher, the editors and the reviewers. Any product that may be evaluated in this article, or claim that may be made by its manufacturer, is not guaranteed or endorsed by the publisher.

## Supplementary Material

Supplementary Figure 1 *Ex vivo* sensitivity of PBMCs to venetoclax or AMG-176 from patients obtained during ibrutinib and venetoclax therapy. PBMCs from one patient were incubated for 24 hours with either DMSO, 100 nM AMG-176, 300 nM AMG-176, 100 nM venetoclax, and combination of AMG-176 with venetoclax at 100 or 300 nM. Cell death was determined using flow cytometry after Annexin V/propidium iodide (PI) staining. AMG, AMG-176 and VEN, venetoclax. C, cycle and D, days. Such incubations were done in 10-15 patient samples and data are presented in **Figure 3**.

Supplementary Figure 2 Changes in the BCR signaling pathway and Bcl-2 proteins during ibrutinib and venetoclax therapy. PBMCs from Pt #0216 were obtained at the indicated times during therapy. Proteins were extracted and immunoblots were performed for BCR signaling pathway proteins and for Bcl-2 family proteins. Similar assays were done for 13-15 patients and such immunoblots were performed for all patients and data were used to quantitate changes during therapy. GAPDH was used as a loading control. C, cycle and D, days.

Supplementary Figure 3 Changes in BCR signaling pathway and Bcl-2 proteins during ibrutinib and venetoclax therapy. PBMCs from Pt #214 were obtained at the indicated times during therapy. Proteins were extracted and immunoblots were performed for BCR signaling pathway and for Bcl-2 family proteins. These were quantitated and plotted compared to baseline (C1D1) sample. Similar samples were collected for 13-15 patients, immunoblots were performed, and data were used to quantitate changes during therapy. GAPDH was used as a loading control. C, cycle and D, days.

Supplementary Figure 4 Changes in BCR signaling pathway and Bcl-2 proteins during ibrutinib and venetoclax therapy. PBMCs from Pt #888 were obtained at the indicated times during therapy. Proteins were extracted and immunoblots were performed for BCR signaling pathway proteins and for Bcl-2 family proteins. These were quantitated and plotted compared to baseline (C1D1) sample. **(A)**. BCR pathway proteins. **(B)**. Bcl-2 anti-apoptotic proteins. **(C)**. Bcl-2 pro-apoptotic proteins. GAPDH was used as a loading control. C, cycle and D, days.

Supplementary Figure 5 Violin plot for changes in the level of phospho- PLCγ2 during therapy. PBMCs from 13-15 patients were obtained at indicated times during therapy. Proteins were extracted and immunoblots were performed for pPLCγ2 at Tyr1252 which is a BTK independent site. Protein levels were quantitated and normalized with GAPDH and compared with the levels at baseline (C1D1). Each symbol represents a patient sample and horizontal lines are median values. Compared to C1D1 levels, the p values for pPLCγ2 are 0.0614, 0.0692, 0.2336, and 0.0436 at C2D1, C4D1, C4D8 and C5D1. C, cycle and D, days

Supplementary Figure 6 Changes in Mcl-1 protein during ibrutinib and venetoclax therapy. PBMCs from 16 patients were obtained at the indicated times during therapy; each symbol represents a patient. Proteins were extracted and immunoblots were performed for Mcl-1 anti-apoptotic protein. These were quantitated, normalized with loading control, and plotted compared to baseline (C1D1) sample. GAPDH was used as a loading control. C, cycle and D, days.

Supplementary Figure 7 Violin plot for changes in the level of PARP protein during therapy. PBMCs from 13-15 patients were obtained at indicated times during therapy. Proteins were extracted and immunoblots were performed for PARP. Protein levels were quantitated and normalized with GAPDH and compared with the levels at baseline (C1D1). Each symbol represents a patient sample and horizontal lines are median values. Compared to C1D1 levels, the p values for PARP are 0.3234, 0.2570, 0.0005, and < 0.0001 at C2D1, C4D1, C4D8 and C5D1. C, cycle and D, days.
