# Supplementary material for: Targeting Mcl-1 by AMG-176 During Ibrutinib and Venetoclax Therapy in Chronic Lymphocytic Leukemia
Source: Front Oncol. 2022 Feb 22;12:833714. doi: 10.3389/fonc.2022.833714 (PMC8901605; doi:10.3389/fonc.2022.833714)
Supplement: Supplementary file 1 [file DataSheet_1.pdf]

Supplemental Table 1. Antibodies for immunoblots and their sources

Antibodies	Source	Catalog #
Anti-Bax	Sigma-Aldrich, St. Louis, MO	B8554
Anti-BAK, NT	EMD Millipore Corporation, Bedford, MA	06-536
Bcl-2	Dako, Carpinteria, CA	M0887
Bcl-XL	Cell Signaling Technology, Danvers, MA	2764S
BCL2A1 (BFL-1)	Origene Technologies Incorporated, Rockville, MD	OTI3D10
BCL2L13 (BCL-2 Rambo)	Proteintech Group, INC., Rosemont, IL	16612-1-AP
BIM	Cell Signaling Technology, Danvers, MA	2933S
Purified Mouse -Anti-Human, Clone 53/BTK BTK	BD Biosciences, San Jose, CA	611117
GAPDH	GeneTex International Corporation, Irvine, CA	GTX627408
Mcl-1	Cell Signaling Technology, Danvers, MA	94296
NOXA	EMD Millipore Corporation, Bedford, MA	OP180
p44/42 MAPK (ERK 1/ 2) (3A7)	Cell Signaling Technology, Danvers, MA	9107S
PARP-1	Enzo Life Sciences, Farmingdale, NY	BML-SA250
Phospho-p44/42 MAPK (ERK 1/ 2) (3A7)	Cell Signaling Technology, Danvers, MA	4370S
Phospho-BTK	Cell Signaling Technology, Danvers, MA	5082S
Phospho-PLCγ2 (Tyr759)	Cell Signaling Technology, Danvers, MA	3874S
Phospho-PLCγ2 (Y1217)	Cell Signaling Technology, Danvers, MA	3871S
PLCγ2	Santa Cruz, Dallas, Texas	sc-5283
PUMA	Abcam LTD, Cambridge, MA	ab33906
Vinculin	Sigma-Aldrich, St. Louis, MO	13901
