# Supplementary material for: Targeting Mcl-1 by AMG-176 During Ibrutinib and Venetoclax Therapy in Chronic Lymphocytic Leukemia
Source: Front Oncol. 2022 Feb 22;12:833714. doi: 10.3389/fonc.2022.833714 (PMC8901605; doi:10.3389/fonc.2022.833714)

**Supplemental Figure 1**

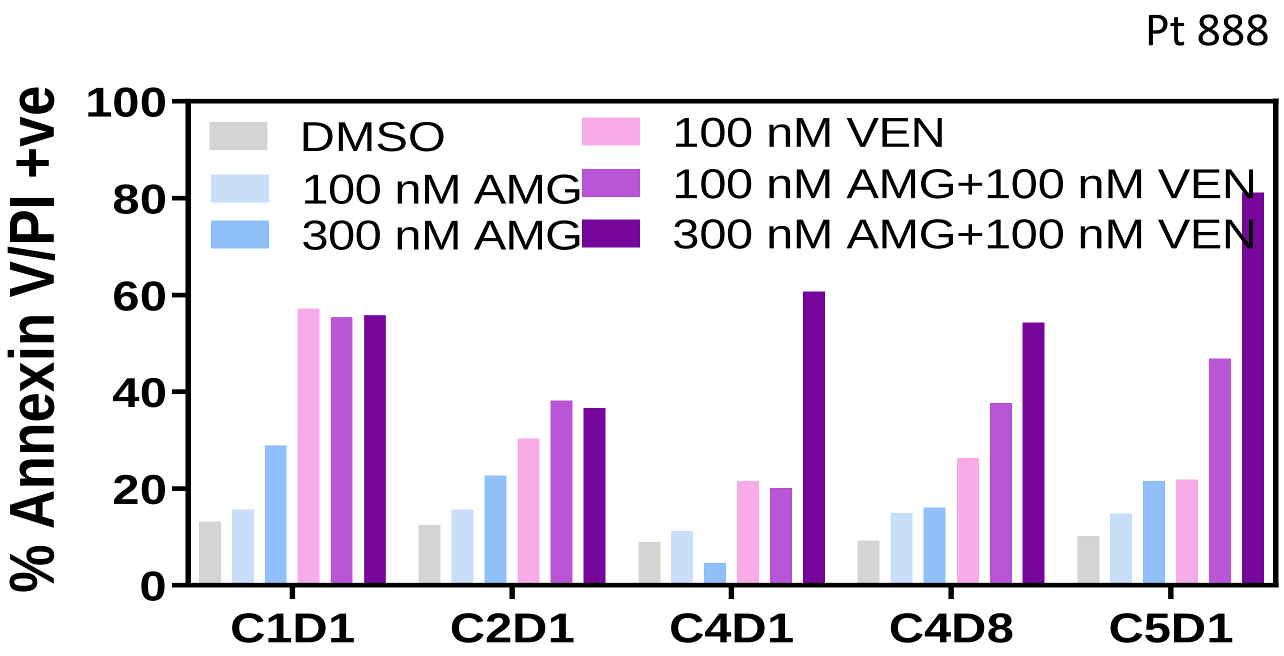

# Supplemental Figure 2

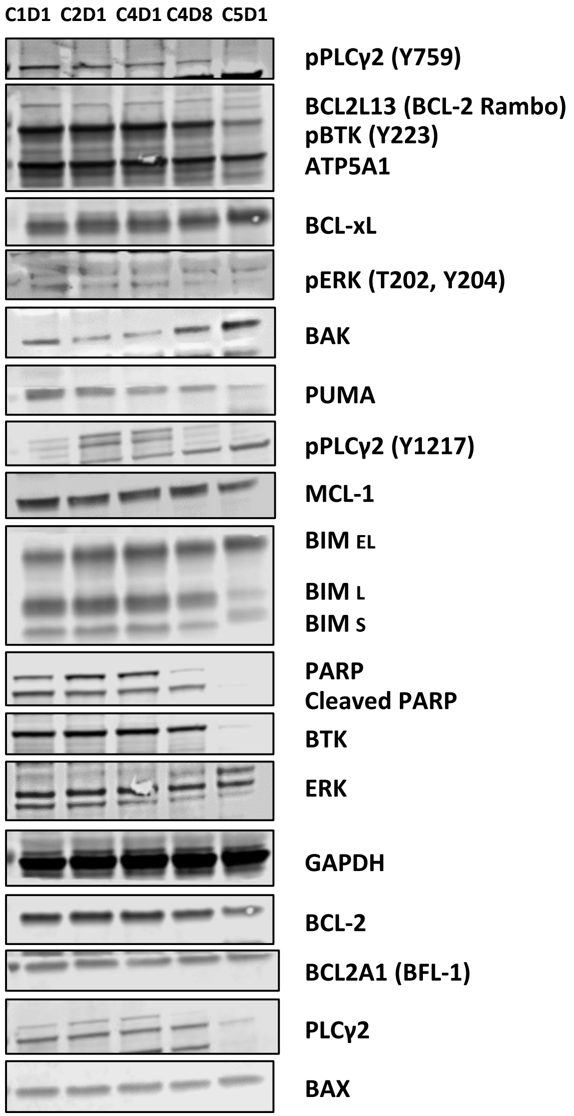

**Supplemental Figure 3**

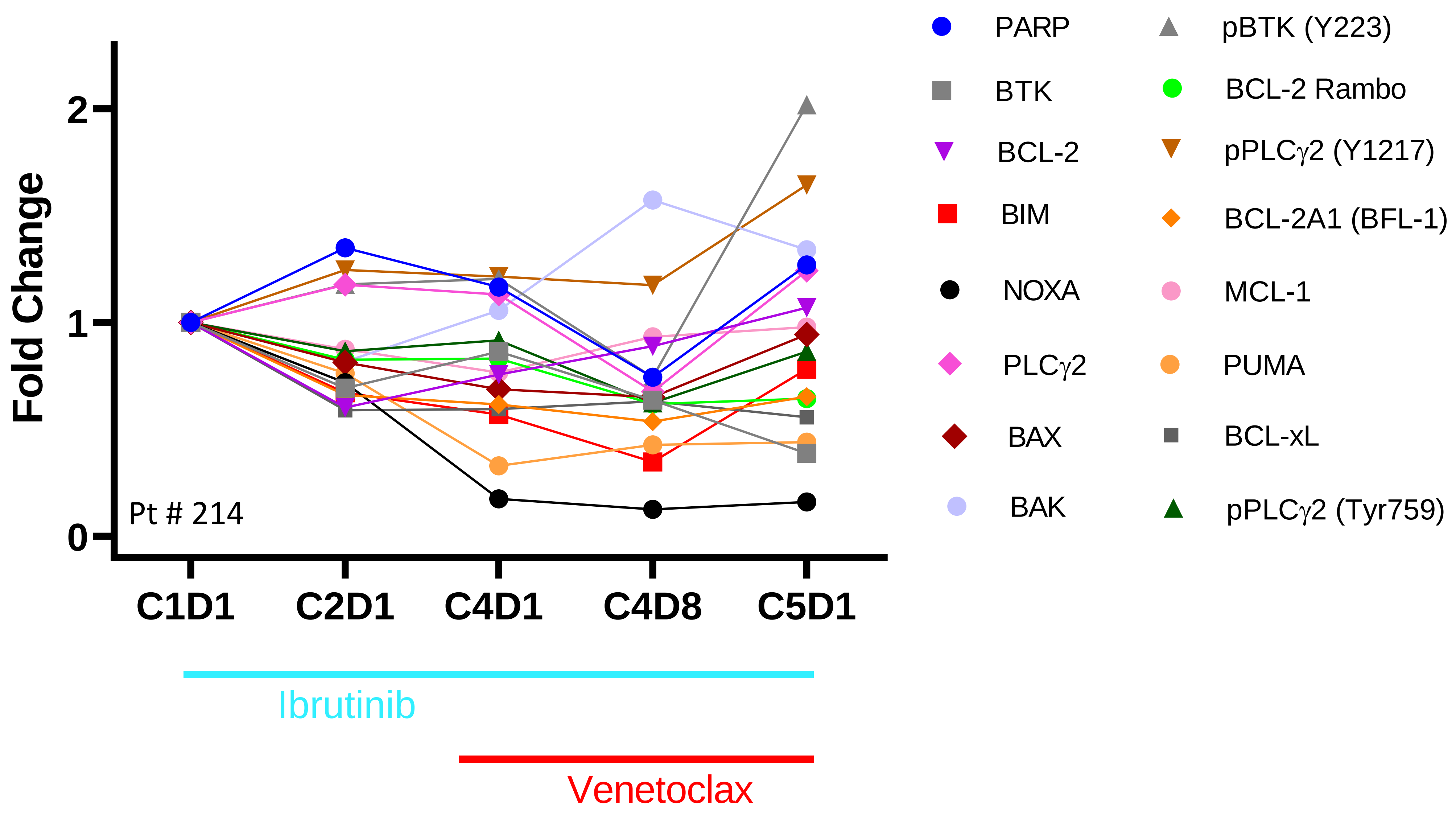

# Supplemental Figure 4

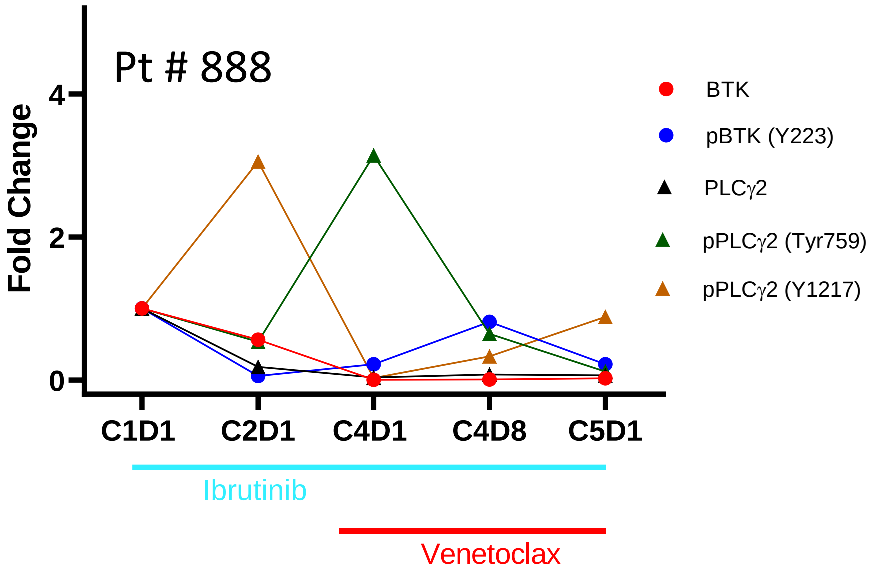


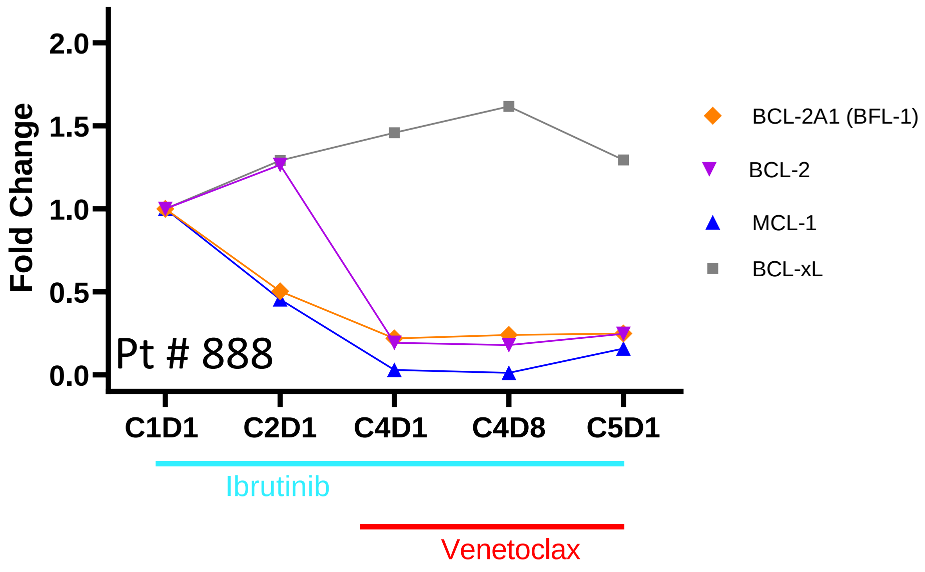


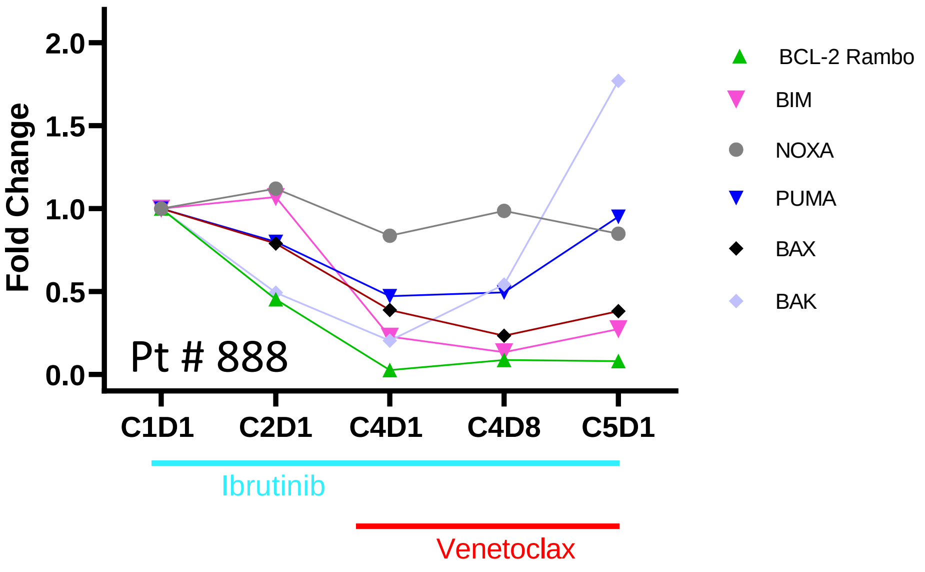

**Supplemental Figure 5**

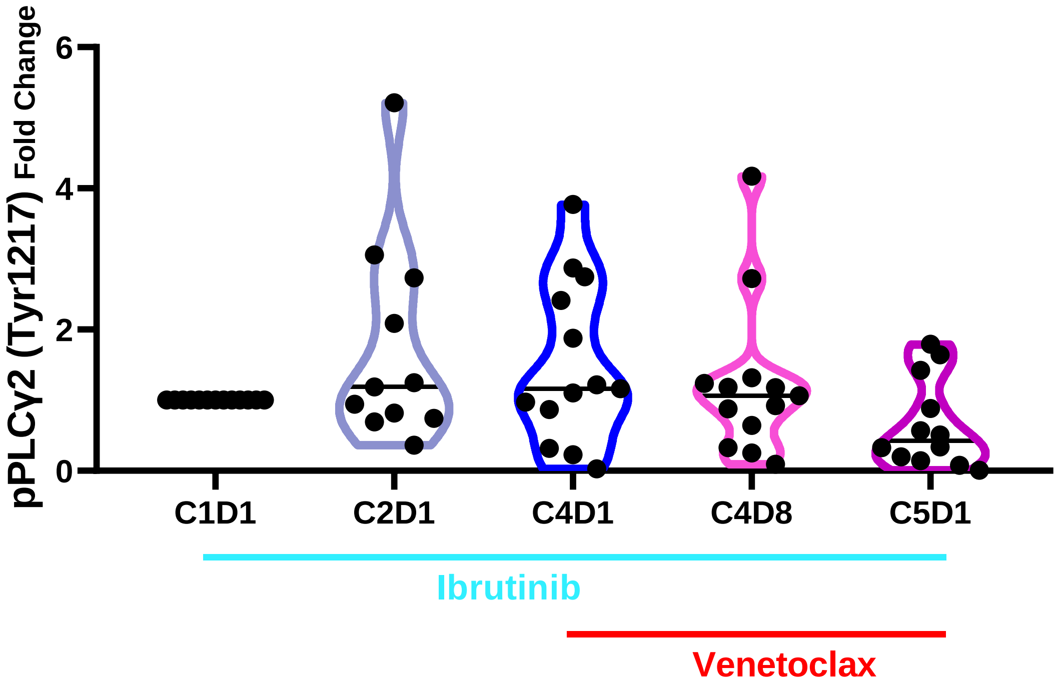

**Supplemental Figure 6**

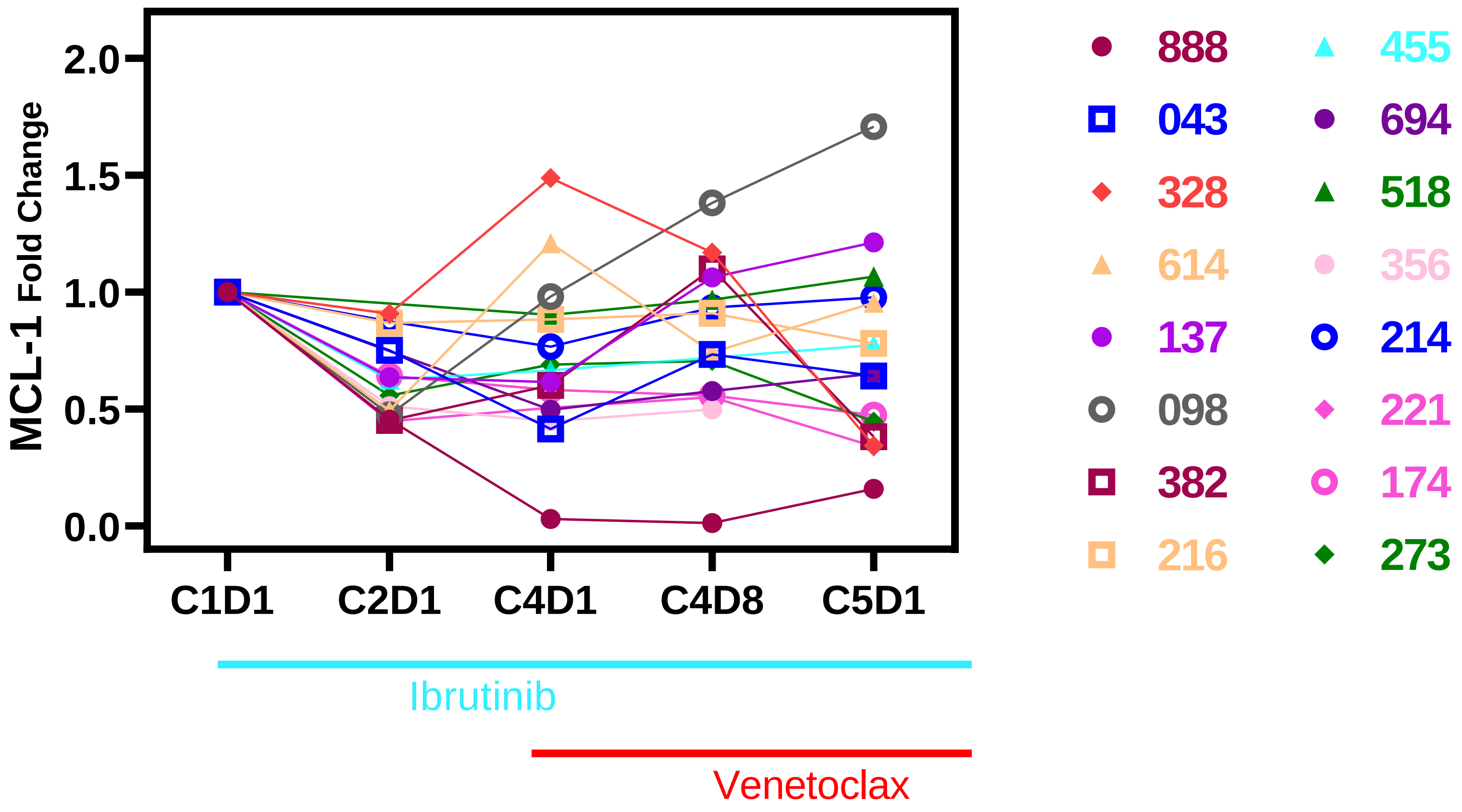

**Supplemental Figure 7**

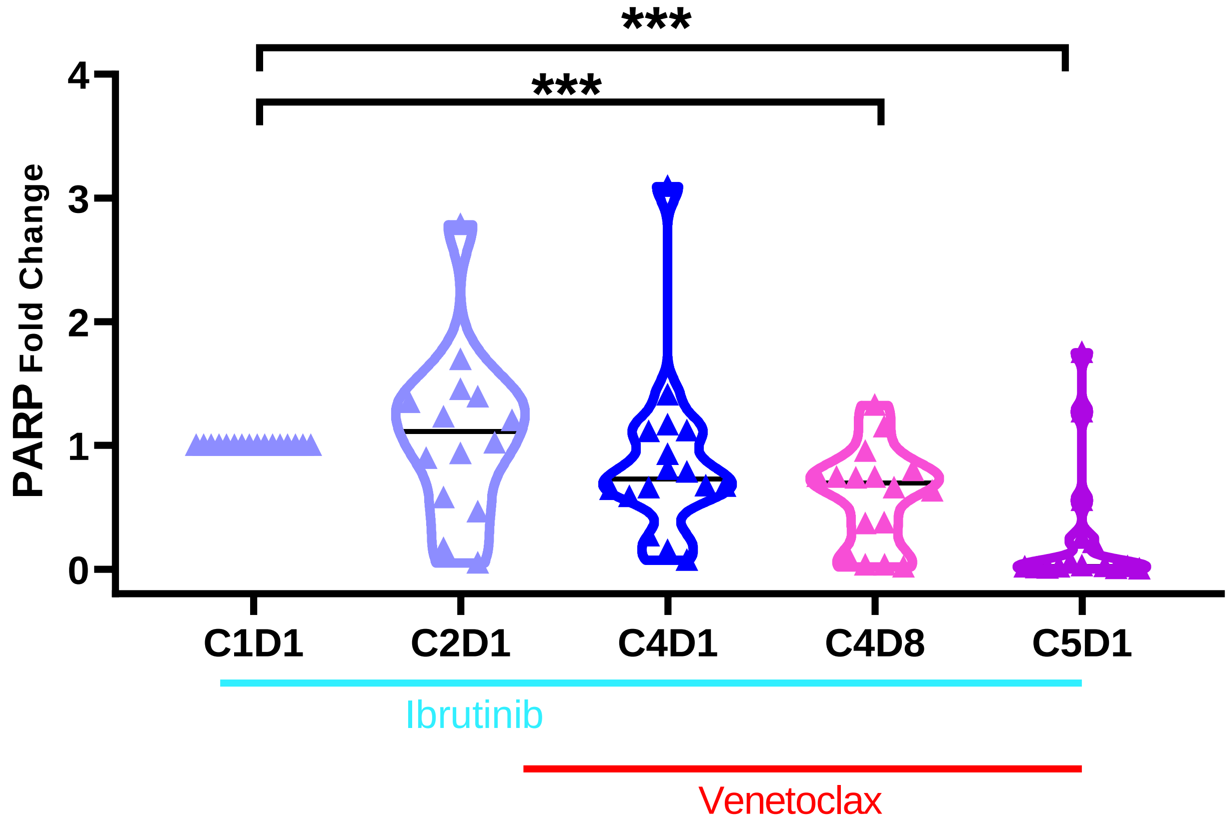

Supplement: Supplementary Figure 1 — Ex vivo sensitivity of PBMCs to venetoclax or AMG-176 from patients obtained during ibrutinib and venetoclax therapy. PBMCs from one patient were incubated for 24 hours with either DMSO, 100 nM AMG-176, 300 nM AMG-176, 100 nM venetoclax, and combination of AMG-176 with venetoclax at 100 or 300 nM. Cell death was determined using flow cytometry after Annexin V/propidium iodide (PI) staining. AMG, AMG-176 and VEN, venetoclax. C, cycle and D, days. Such incubations were done in 10-15 patient samples and data are presented in Figure 3. [file DataSheet_2.pdf]
